# From Mollusks to Medicine: A Venomics Approach for the Discovery and Characterization of Therapeutics from Terebridae Peptide Toxins

**DOI:** 10.3390/toxins8040117

**Published:** 2016-04-19

**Authors:** Aida Verdes, Prachi Anand, Juliette Gorson, Stephen Jannetti, Patrick Kelly, Abba Leffler, Danny Simpson, Girish Ramrattan, Mandë Holford

**Affiliations:** 1 Hunter College, The City University of New York, Belfer Research Building, 413 E. 69th Street, New York, NY 10021, USA; averdes@gradcenter.cuny.edu (A.V.); panand@hunter.cuny.edu (P.A.); jgorson@gradcenter.cuny.edu (J.G.); sjannetti@gradcenter.cuny.edu (S.J.); mkelly3@gradcenter.cuny.edu (P.K.); ael355@nyu.edu (A.L.); cs4379@nyu.edu (D.S.); girish.ramrattan22@myhunter.cuny.edu (G.R.); 2 The Graduate Center, City University of New York, 365 5th Ave, New York, NY 10016, USA; 3 Sackler Institute for Comparative Genomics, Invertebrate Zoology, American Museum of Natural History, Central Park West & 79th St, New York, NY 10024, USA; 4 Sackler Institute of Graduate Biomedical Sciences, New York University School of Medicine 550 1st Avenue, New York, NY 10016, USA; 5 Tandon School of Engineering, New York University 6 MetroTech Center, Brooklyn, NY 11201, USA

**Keywords:** venomics, Terebridae, teretoxins, peptide toxins, animal venom, venom peptides, drug development, drug discovery, peptide therapeutics, drug delivery

## Abstract

Animal venoms comprise a diversity of peptide toxins that manipulate molecular targets such as ion channels and receptors, making venom peptides attractive candidates for the development of therapeutics to benefit human health. However, identifying bioactive venom peptides remains a significant challenge. In this review we describe our particular venomics strategy for the discovery, characterization, and optimization of Terebridae venom peptides, teretoxins. Our strategy reflects the scientific path from mollusks to medicine in an integrative sequential approach with the following steps: (1) delimitation of venomous Terebridae lineages through taxonomic and phylogenetic analyses; (2) identification and classification of putative teretoxins through *omics* methodologies, including genomics, transcriptomics, and proteomics; (3) chemical and recombinant synthesis of promising peptide toxins; (4) structural characterization through experimental and computational methods; (5) determination of teretoxin bioactivity and molecular function through biological assays and computational modeling; (6) optimization of peptide toxin affinity and selectivity to molecular target; and (7) development of strategies for effective delivery of venom peptide therapeutics. While our research focuses on terebrids, the venomics approach outlined here can be applied to the discovery and characterization of peptide toxins from any venomous taxa.

## 1. Introduction

Medicinal treatments have a storied history tied to natural products discovery and development. Natural products derived from plants and animals have been the source of traditional medicine for millennia, and more recently have become major sources of chemical diversity as drug leads, driving research efforts in pharmaceutical drug discovery and development [1,2]. The ascendancy of natural products was acknowledged with the awarding of the 2015 Nobel Prize in Physiology or Medicine for the discovery of two revolutionary therapies based on natural compounds, Avermectin and Artemisinin. Avermectin has helped to nearly eradicate parasitic worm diseases such as river blindness and lymphatic filariasis, while Artemisinin represents the most effective treatment for malaria known to date [3]. The impact of these natural products on improving global human health is incalculable.

**Figure 1:**
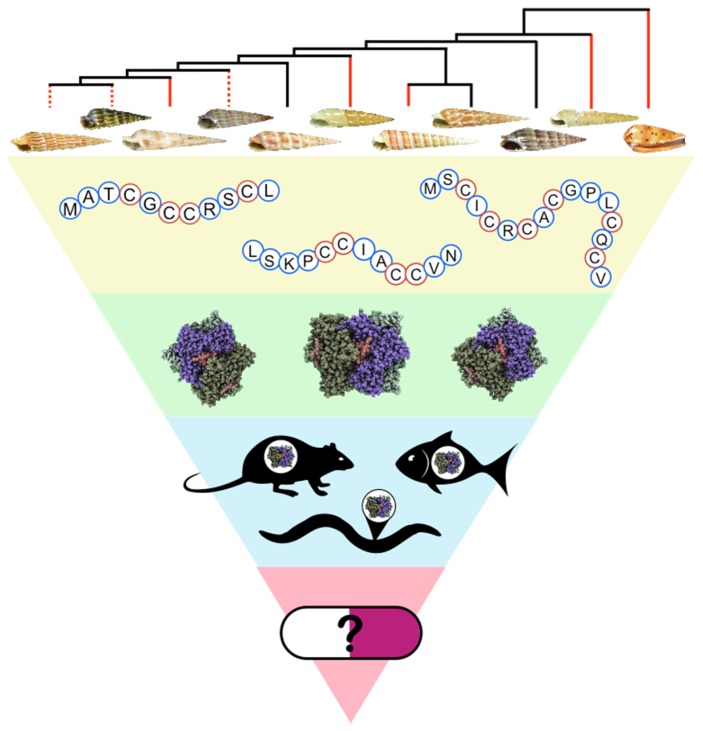
From mollusks to medicine. Overview of venomics approach for discovery, characterization, and development of therapeutics from Terebridae venom peptides. This strategy begins with a phylogenetic delimitation of venomous terebrid lineages to identify the species that are producing venom to subdue their prey (shown in red); in yellow, identification of teretoxins through *omics* (genomics, transcriptomics, proteomics); in green, synthesis and structural characterization of teretoxins; in blue, bioactivity assays and identification of molecular targets; and in pink, peptide optimization and development of delivery methods for potential terebrid therapeutics.

The journey from natural product discovery to therapy has largely focused on small chemical compounds such as Avermectin and Artemisinin; however, natural peptides are increasingly being investigated as drug leads in pharmaceutical research [4]. In particular, peptides found in venomous organisms are a very promising source for drug discovery. Successful examples of drugs developed from venom peptides include Captopril^®^, based on a venom peptide from the Brazilian viper and used to treat hypertension [5,6]; exenatide (marketed as Byetta^®^), based on the Gila monster venom and used as an anti-diabetic agent [7]; and ziconotide (Prialt^®^), based on a venom peptide from the predatory cone snail *Conus magus* and used to treat chronic pain [8,9]. Most venom peptides are disulfide-rich and vary in length from 12–30 residues in cone snails to 40–80 residues in terebrids, scorpions, and snakes [10,11,12]. The relatively small size and the stability provided by disulfide bridges that characterize natural peptides make them ideal candidates for drug leads. Venom peptides are predominantly being investigated for the development of drug therapies targeted to ion channels and receptors [12,13,14,15,16]. Due to technological constraints, such as size and ease of collection, venomous organisms like snakes and scorpions have been traditionally singled out for drug discovery research. However, recent advances in next-generation sequencing (NGS) techniques and improvements in proteomic methods have allowed venom research to expand and include neglected venomous invertebrates with great potential, such as the conoideans (Figure 1) [17,18,19].

The Conoidea superfamily (cone snails, terebrids, and turrids *s.l.*) is an extremely diverse group of predatory marine neogastropods divided into 16 families, with several lineages characterized by having a venom apparatus used for predation [10,19,20]. The genus *Conus*, the most extensively studied among the conoideans, and from which the drug ziconotide (Prialt^®^) was discovered, includes species that produce very complex venoms with thousands of unique venom peptides, known as *conotoxins or conopeptides* [21,22,23,24,25,26,27]. As such, it is not surprising that conotoxins have been considerably studied for several decades. However, the ~700 described species of cone snails represent far less than half of the over 15,000 species that are estimated to comprise the *Conoidea* superfamily [28]. The family Terebridae, commonly known as auger snails, is an understudied lineage of conoideans that also has venomous representatives [29,30,31,32,33].

There are circa 400 described species of terebrids that live mostly in shallow sandy bottoms on tropical waters and have a characteristic elongated shell [33,34,35]. Terebrid venom peptides, referred to as *teretoxins*, are structurally similar to conotoxins, but due to the early divergence of terebrids and cone snails in the Paleocene [36], teretoxins represent highly divergent compounds with unique functionalities compared to conotoxins [10,19,37,38,39].

Despite their great potential, characterizing bioactive compounds in conoidean venom poses several challenges due mainly to their great species diversity, difficulty of sampling due to size and habitat, the small amounts of venom produced, and the scarcity of reference databases to identify novel venom peptides [40]. The most promising avenue to overcome these challenges is to apply interdisciplinary strategies that integrate molecular biology and biochemical analyses of venom compounds to optimize the characterization of peptides [41]. This strategy, often referred to as *venomics*, combines classic approaches for the study of biodiversity, such as taxonomy and phylogeny, with modern NGS techniques and proteomic methods, creating a robust evolutionary roadmap for effective drug discovery while greatly advancing knowledge on venom systematics and evolution [16,19,42,43,44].

In the present review, we describe our specific venomics approach to investigate Terebridae diversity and evolution, and to identify and characterize teretoxins and their potential for biomedical applications, paving the scientific route from mollusks to medicine (Figure 1).

## 2. Phylogeny-Based Discovery of Teretoxins

**Figure 2:**
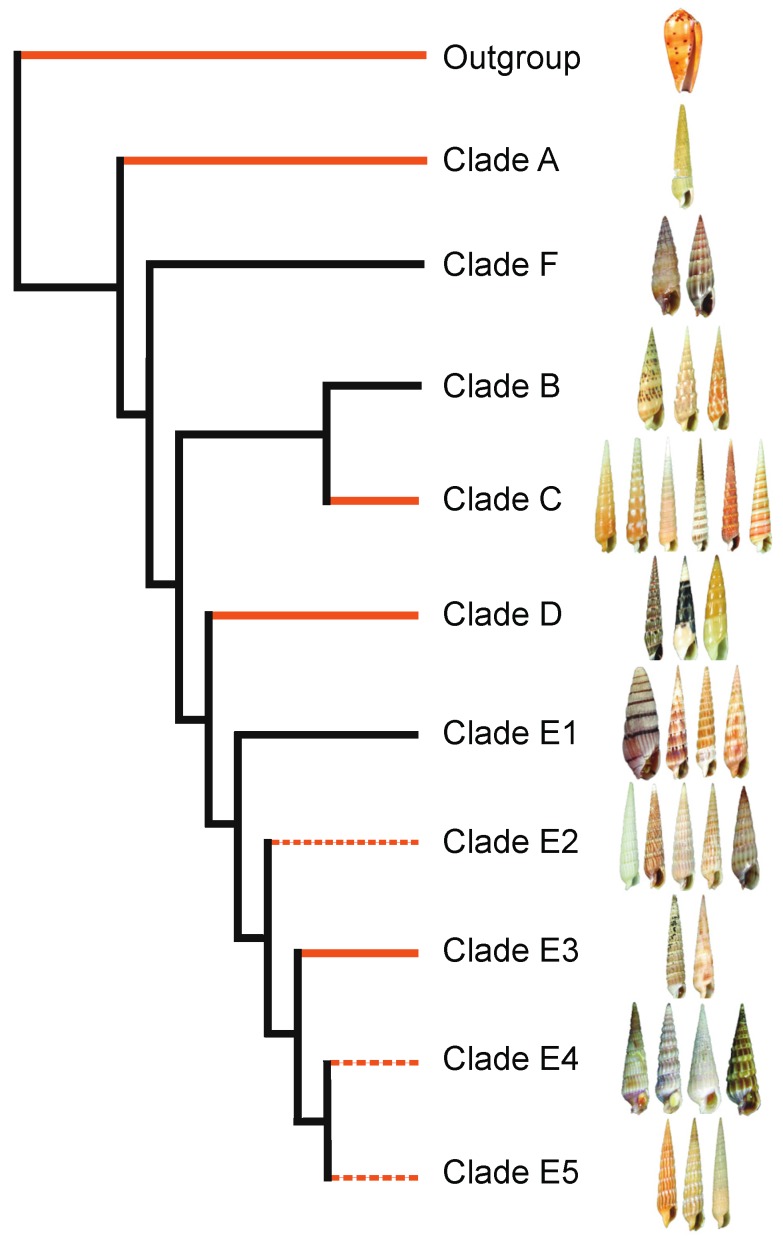
Terebridae phylogeny. Cladogram reconstructing the evolutionary relationships within the Terebridae family. Line color indicates presence or absence of venom apparatus. Solid red lines indicate clades in which all members have venom apparatus, dashed red lines indicate clades in which only some members have venom apparatus, and black lines indicate clades that lack venom apparatus. Cladogram based on phylogenetic reconstruction from [48].

Traditionally molluscan species were chosen for venom research based on size, ease of collection, and quantity of venom produced. The lack of a methodological strategy led to the characterization of random venoms that sometimes corresponded to a mere single lineage [19]. As molecular biology, NGS, and proteomics technologies advanced, size and quantity of venom were no longer a restriction and it was possible to devise strategies that harnessed the evolutionary power of nature, investigating phylogenies and species relatedness to determine the most promising and diverse conoidean lineages to identify novel bioactive compounds through venomics analyses [22,45,46,47]. This venomics-based discovery strategy takes into account different characteristics, such as the presence of a venom apparatus, and demonstrates the importance of understanding phylogeny to enhance the identification of venom peptides with potential pharmacological applications [19]. We follow this phylogeny-informed methodology to select appropriate terebrid study lineages and taxa (Figure 2) [10].

### 2.1. Terebridae Phylogenetics

Natural history and relatedness among species have been traditionally defined by morphology-based phylogenetic reconstructions. This methodology is hampered in the Neogastropoda due to the high levels of homoplasy and convergence in morphological characteristics [49,50,51]. Thus, the advantages of molecular phylogenetics, which allows for the comparison of thousands of homologous characters across species, are of particular interest among the Terebridae.

The first molecular phylogeny of the Terebridae was constructed based on analyses of a three-gene matrix (12S, 16S, and COI) to define Terebridae lineages and their evolutionary history [52]. This initial Terebridae phylogeny confirmed the monophyly of the group and defined five distinct lineages: *Acus* (Clade B), *Terebra* (Clade C), *Hastula* (Clade D), *Myurella* (Clade E), and a previously unidentified fifth sister clade that includes *Pellifronia jungi* (Clade A) [52]. Subsequent molecular phylogenetic analysis, including additional taxa from the Eastern and Western Pacific further resolved the terebrid evolutionary relationships, synonymizing *Acus* clade B to *Oxymeris*, recovering a previously unidentified clade F that includes the *Euterebra* and *Duplicara* genera, and subdividing the large *Myurella* clade E into five lineages (Clades E1–5) [48] (Figure 2). The molecular phylogeny of terebrids correlates with anatomical features, specifically the presence or absence of the venom apparatus [53].

It is important to mention that the accuracy of phylogenetic reconstructions is not guaranteed by any particular number of genes or taxa, even when bootstrap support values are high. In many cases, increasing gene number leads to higher support for the incorrect phylogenetic reconstruction; however, increasing taxon representation improves the accuracy, providing a phylogeny that is more likely to represent the evolutionary history of the group. Therefore, the accuracy of phylogenetic estimations as well as the accuracy of inferences about evolutionary processes based on phylogenies can be significantly improved by extensive and thorough taxon sampling efforts [54,55]. This was evident in the last Terebridae phylogeny published in 2012, which expanded the taxon sampling from the Western Pacific region to include species from the Eastern Pacific as well. The expansion of taxon sampling allowed us to substantially refine the relationships of the Myurella clade lineages and to recover a previously unidentified clade F (Figure 2) [48,52]. For this reason, we are constantly working on increasing taxon sampling to improve the phylogenetic reconstruction of the Terebridae and currently have samples of ~150 species, which represents ~38% of the 400 currently known terebrid taxa.

Another source of conflict when inferring phylogenies is determining the root of the tree. The root of a tree represents its deepest split and determines the direction of all subsequent evolutionary events. An incorrect root can result in erroneous inferences of species relationships and character evolution and, therefore, determining the root accurately is critical for phylogenetic analysis. One of the most common methods applied to root phylogenetic trees is the use of an outgroup that represents the most closely related taxa or sister group. Unfortunately, it is not always certain what the closest relatives to a particular group are and, even when this is known, sometimes the closest relatives are rather distantly related [56,57]. Luckily, the Conoidean phylogeny has been thoroughly studied and there is extensive evidence that cone snails and turrids are the most closely related taxa to Terebridae [20,58]. Therefore, the Terebridae phylogenetic reconstructions are rooted with representative species of Conidae and Turridae as closely related outgroups and species from the neogastropod family Harpidae as distant outgroups [52], providing a robust and accurate root for phylogenetic inference.

### 2.2. Venom Apparatus Evolution

The venom apparatus as defined in the Conidae consists of a venom bulb, a venom gland, a radular sac, and a proboscis. However, the Terebridae have been traditionally described as having three distinct foregut anatomies: (I) salivary glands present, but lack of a radular sac and venom apparatus; (II) identical to a *Conus* venom apparatus with a radula delivery system and venom gland for venom production; and (III) lack of a venom apparatus, but presence of an accessory proboscis structure [59,60]. These early anatomical descriptions have been revised in recent publications and expanded to include additional important features of terebrid anatomy, such as marginal radular teeth [61,62,63]. Terebrids display the widest diversity of marginal radular teeth types in all conoideans including duplex, solid recurved, flat, semi-enrolled, and hypodermic. These teeth are absent in the lineages in which the venom apparatus has been lost [48]. Our recent efforts have also revealed that the evolution of the Terebridae foregut anatomy is rather complex and certain features have originated independently across the phylogeny, while others including the proboscis, radula, and venom gland have been lost in several lineages [48]. The venom gland specifically, was lost eight times throughout Terebridae evolution in clades F, B, and E1, and in certain members of E2, E3, E4, and E5 [48]. This level of gain and loss of venom-related characters is similar to what has been observed in other venomous taxa such as fish, lizards, and snakes [64,65,66].

The morphological diversity of foregut anatomies in the Terebridae is hypothesized to correlate with the varying diet and feeding strategies among the different terebrid lineages, and it has been suggested as one of the main drivers of species diversification in the group. Moreover, terebrids with the Type II feeding apparatus feed on their prey in a manner that mirrors that of cone snails. Specifically, *Hastula* and *Terebra* species use a hypodermic radular tooth at the end of the proboscis to envenomate their vermivorous prey [32,34,48,59,67,68]. Venom variability in cone snails has been extensively studied and the differences in peptide diversity and expression patterns among different species have been attributed to divergent diets and defensive pressures, which in turn drive species diversification [69,70,71]. Consequently, we can expect a similar correlation pattern to that of cone snails, with increased species numbers in the Terebridae lineages that have venom apparatus, and, accordingly, a vast diversity of terebrid venom peptides.

As the venom apparatus is not found in all terebrid lineages, the first step to characterize teretoxins is to successfully identify the lineages that have a venom gland and are actively expressing venom peptides to subdue their prey or for defensive purposes. The molecular phylogeny and characterization of terebrid foregut anatomy completed to date provides a roadmap for efficiently identifying the most promising terebrid lineages for venomics investigation (Figure 2). Understanding the relationships between terebrid lineages aids in effectively identifying divergent terebrid groups for the discovery of novel peptides with diverse molecular activities that can be used to further drug discovery research.

## 3. Teretoxin Identification and Classification

The traditional approach for peptide toxin discovery employed biochemical techniques such as venom fractionation by Liquid Chromatography (LC), Edman Degradation to determine primary amino acid sequences, and Mass Spectrometry (MS) to characterize crude venom extracts. However, with the decreasing costs and increasing efficiency of NGS techniques and improvements in high-throughput proteomic methods, the venomics landscape is rapidly changing and currently even organisms that produce exceptionally small quantities of venom can be characterized [72]. Transcriptomic studies of venom duct and venom gland tissue are rapidly growing for a number of venomous taxa, providing large amounts of data that allow the analysis of expressed gene products and the identification of a great number of putative peptide toxins [73,74,75,76,77]. However, these studies also have disadvantages. For example, venom peptides identified by genomic methods cannot be validated without proteomic evidence [78,79,80,81,82,83]. Conveniently, modern technologies allow the use of sequence databases generated from genomic data or available in public databases such as Conoserver and Tox-Prot to aid in the identification of peptides from proteomic data [84,85,86,87]. Additionally, the large number of putative venom peptides and proteins identified by NGS and high-throughput proteomics can be classified into gene superfamilies using phylogenetic methodologies to facilitate their interpretation and assist with functional predictions [10,25].

### 3.1. Venom Gland Transcriptomics

**Figure 3:**
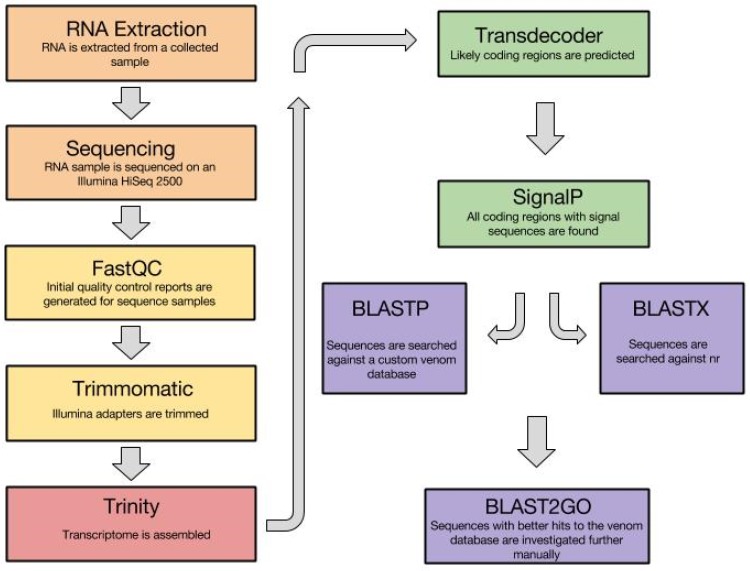
Bioinformatics pipeline for terebrid transcriptome analyses. Summary of bioinformatics pipeline for the identification of putative teretoxins from RNA-Seq data. Colors indicate different stages of the process. Orange indicates RNA extraction and sequencing, yellow indicates raw read quality filtering, red indicates transcriptome assembly, green indicates ORF and signal sequence prediction from transcripts, and purple indicates transcript annotation.

Venom gland transcriptome studies have proven very useful to characterize putative venom compounds in small invertebrates such as the Terebridae. We have taken advantage of these methods and recently published a comparative analysis of the venom gland transcriptomes of two Terebridae species, providing important insights into terebrid venom composition and evolution [10]. In this work we developed an *in silico* bioinformatics pipeline that can be broadly applied to investigate transcriptomic data from other venomous organisms (Figure 3). The pipeline begins offline with collection of species and tissue dissections. Specifically, terebrid specimens are collected from tropical marine habitats and dissected to extract the venom gland, which is flash frozen in liquid nitrogen or fixed in RNA*later* and stored at −80 °C until ready for use. For our purposes, total RNA is extracted from venom gland tissue with the Qiagen RNeasy Micro Kit following the manufacturer protocol and sequenced using Illumina HiSeq 2500 with v. 4 technology using a paired end flow cell and 100 × 2 cycle sequencing.

The quality of the raw Illumina sequence reads is then evaluated with FastQC (http://www.bioinformatics.babraham.ac.uk/projects/fastqc). FastQC generates a profile of sequencing data, including graphs of quality per base, GC-content, k-mer content, and sequence length distributions among others, allowing for a quick assessment of potential sequencing errors [88]. Trimmomatic [89] is subsequently used to trim poor quality reads and to remove any Illumina adapters present and the processed reads are assembled *de novo* using Trinity [90,91]. Using Trimmomatic to remove low-quality reads can lead to a higher quality assembly, but the assembly itself and all putative venom peptides identified must be treated with caution due to the lack of a reference terebrid genome and the complexity of assembling hypervariable venom peptides, which can be a challenge for existing assembly software programs.

After transcriptome assembly is completed, TransDecoder is used to predict coding regions within the transcripts. A sequence is classified as a candidate protein-coding region based on nucleotide composition, open reading frame (ORF) length, and optionally, a match to a Pfam domain [90]. As venom is mainly composed of secreted proteins and peptides, SignalP is then used to predict signal peptide sequences in these putative protein-coding regions [92]. Using a custom Perl script, all transcripts surviving these two initial filters are then searched against an in-house venom database using the BLASTp tool [93]. This database includes all known venom proteins and peptides available in public databases such as Conoserver and Tox-Prot along with putative teretoxins identified by our group [85,86,87]. All transcripts with hits to a protein in the database with an e-value of 1e–5 or better and sequence similarity of at least 40% are then searched against the NCBI non-redundant (nr) database using the BLASTx tool with the same e-value and sequence similarity thresholds. The results from the two BLAST searches are compared, and those with a better hit to a protein in the venom database are considered putative teretoxins and further investigated. The high variation present in venoms makes identification via homology comparison potentially error-prone, with a high number of false positive predictions. Without verification via experimental techniques such as mass spectrometry, the actual existence of predicted teretoxins from our pipeline cannot be determined with certainty.

The amino acid sequences of putative teretoxins are also processed for mapping, annotation, and, specifically, the assignment of Gene Ontology (GO) terms in BLAST2GO [94,95]. The assignment of GO terms provides information about putative gene or protein domain functions, strengthening the identification of candidate teretoxins. BLAST2GO is also used to identify potential venom peptides when transcripts that encode a signal sequence show no sequence homology to proteins in the venom database through BLAST searches. In this case, protein family IDs and specific protein domains are identified through an automated model-based approach based on InterProScan [96,97]. Following this approach, a carefully curated and annotated final list of candidate teretoxins is generated, allowing the classification of transcripts into functional categories for comparative studies across taxa.

### 3.2. Identification of Teretoxin Superfamilies

Conoidean venom peptides have a characteristic structure, namely, a signal peptide sequence followed by a propeptide region and a terminal cysteine-rich mature peptide. Conotoxins have been classified into “gene superfamilies” according to the percentage of sequence identity of their signal peptide [98]. Venom gene superfamilies are hypothesized to reflect the evolutionary history of the conotoxin multigenic system. Puillandre *et al.* [99] recently validated this hypothesis and provided a phylogenetic framework for the classification of novel conotoxins. With the increasing number of putative conotoxins currently identified though transcriptome sequencing, the phylogenetic classification of conotoxins into venom gene superfamilies facilitates their interpretation and aids in predicting their biological function [24,25].

**Figure 4:**
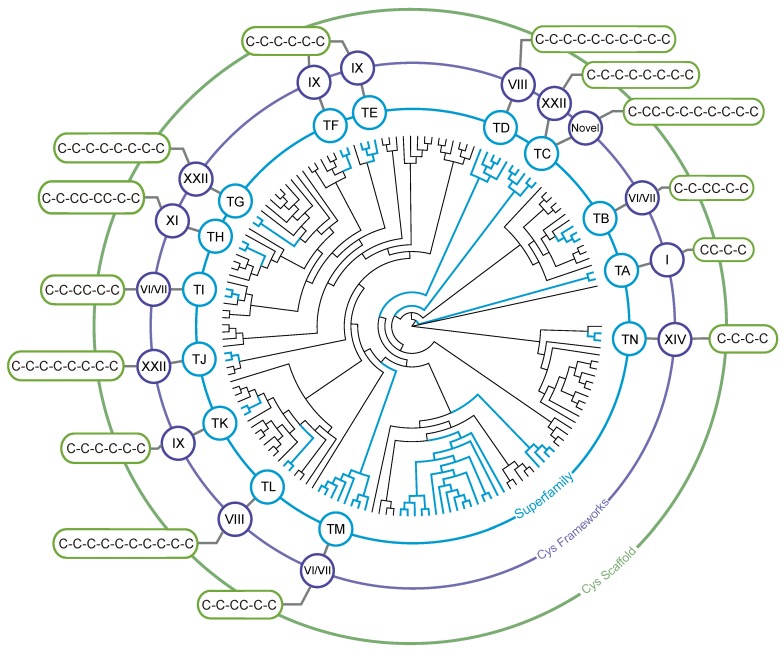
Teretoxin gene superfamilies. Phylogenetic reconstruction of teretoxin gene superfamilies adapted from [10]. Clades representing teretoxin superfamilies are indicated in blue. The cysteine framework that characterizes each superfamily is denoted in purple and the corresponding cysteine scaffold in green. Terebrid superfamily TM is the only one with known homology to a conotoxin superfamily.

Similar to conotoxins, teretoxins are expressed as a single gene product with a signal sequence, propeptide region, and a cysteine-rich mature peptide on the *C*-terminal. While teretoxin gene sequences have been previously reported, there have been no teretoxin gene superfamilies described due mainly to lack of available data [37,38,39]. To address this gap, we recently proposed the first classification of Terebridae teretoxin gene superfamilies, providing a phylogenetic framework for the classification of novel terebrid peptides [10]. Escalating the previous definition used to describe a conotoxin superfamily, we define a teretoxin superfamily using three criteria: (i) independent lineage with high support values (bootstrap ≥70 and posterior probability ≥90); (ii) sequence identity within the superfamily to be greater than or equal to 60%; and (iii) the pattern of cysteines is different than in the sister clade. Through comparative analyses of the venom gland transcriptomes of *Terebra subulata* and *Triplostephanus anilis*, 139 novel putative teretoxins were identified, and following a phylogenetic approach 14 putative terebrid toxin gene superfamilies were described, 13 of which are unique to the Terebridae and thus distinct from any currently known conotoxin superfamilies (Figure 4). The significant differences in the venom profiles of cone snails and terebrids support the premise that the early divergence of the two neogastropod lineages led to distinct venom cocktails [36]. These results illustrate the power of NGS techniques to provide data that can greatly expand venom evolutionary research.

### 3.3. Venom Proteomics and Proteogenomics Analyses

As most NGS bioinformatics pipelines, the one outlined here to analyze terebrid venom gland RNA-Seq data, is heavily reliant on sequence homology searches, thus hindering the ability to identify novel peptide toxins and venom proteins [43]. While there are some computational methods such as InterProScan [96,97] that can aid in the identification of putative venom peptides without sequence homology to known peptide toxins, the presence of the predicted mature peptides in the venom cannot be confirmed without proteomic evidence [43].

The best method to date for the characterization of novel venom peptides is through MS proteomic analyses of venom extracts. Notably, the technology and methodology employed to identify and validate venom peptides via proteomics analyses has vastly changed in recent years. In traditional bottom-up proteomics, enzymatic digestions of venom samples, liquid chromatography (LC), and tandem mass spectrometry (MS/MS) analyses were used to identify venom peptides in a sample. The bottom-up approach can be useful, but due to loss of peptides during purification it can prove unsuccessful at identifying complete sequences, especially when looking for novel peptides. In top-down proteomics, individual intact venom proteins can be characterized and profiled using a direct analysis that compares statistically meaningful numbers in the sample to determine relative expression levels of intact peptides [72,79,100]. While the debate over top-down *versus* bottom-up proteomics continues, top-down has several attractive features for *de novo* venom peptide identification [101]. The top-down approach involves the analysis of intact proteins typically using electrospray ionization and high-resolution mass analysis and is being increasingly used to analyze single proteins or simple protein mixtures, including recent proteovenomic analyses of peptidic and small-protein venoms [72,79,102]. For example, Quinton *et al.* [100] were successful in introducing a rapid top-down sequencing method that used MALDI matrix enhancing in-source decay (ISD) to identify disulfide-bridged peptides in *Conus* venoms. This approach has not only improved the analysis and characterization of animal venoms, but it has also further enabled the identification of post-translational modifications (PTMs) [43,103].

PTMs are very common in conotoxins and can impact their specificity and activity [104,105,106]. The presence of PTMs cannot be reliably inferred from sequence data alone and must be confirmed by MS analysis of the pure native venom extract [47]. For example, proteomic analysis of the venom gland of *Conus textile* identified 31 conotoxins and 25 PTMs, while the venom gland transcriptome analysis of *Conus tribblei* revealed 136 putative conotoxins, and no PTMs [78,107]. While the number of putative conotoxins identified through transcriptomic analyses is much greater, without proteomic evidence none of the 136 *Conus tribblei* putative conotoxins can be validated, nor any potential PTMs identified. Consequently, a combined proteomics–genomics approach, or *proteogenomics,* represents the most comprehensive and promising method for the discovery of novel toxins and the characterization of animal venoms in general, and Terebridae venom in particular [41,43,84]. With this approach, species-specific protein sequence databases generated from genomic and transcriptomic data are used to identify novel peptides, not present in reference databases, from proteomic data. In addition, proteomic data provides evidence of gene expression, validating the gene models predicted from genomic and transcriptomic data. The venom peptides, validated through proteogenomics methods, can then be synthesized and characterized to investigate their function and molecular targets.

## 4. Chemical and Recombinant Peptide Synthesis of Teretoxins

Teretoxins are a valuable reservoir of bioactive compounds; however, due to the scant quantities of venom produced by terebrids, it is difficult to obtain sufficient amounts of venom peptides for appropriate biochemical characterization. This obstacle can be overcome by producing synthetic versions of the peptides found in venom extracts. The three most common ways to obtain venom peptides synthetically are liquid-phase peptide synthesis (LPPS), solid-phase peptide synthesis (SPPS), and recombinant biology techniques [72,108,109]. Each method has advantages and disadvantages, such as the inexpensiveness and simplicity of LPPS that comes at the cost of yield and time. SPPS in turn, offers rapid syntheses, but depending on the peptide, obtaining the native cysteine fold can be problematic [110]. Recombinant synthesis allows for high yield and purity, but does not easily permit the incorporation of unnatural amino acids or site-specific labeling of peptides. The typically small volume of venom produced by terebrids requires multiple synthetic approaches including both chemical and recombinant synthesis methods [72,111].

### 4.1. Solid Phase Peptide Synthesis

**Figure 5:**
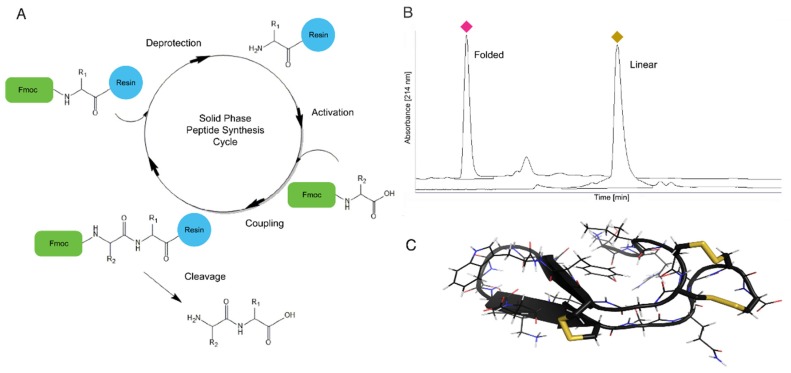
Chemical synthesis of teretoxin Tv1. (**a**) Automated cycle of solid-phase peptide synthesis using FMOC chemistry; (**b**) RP-HPLC chromatogram of Tv1 synthesis (linear) and folding reaction. The folded conformation is indicated by the pink diamond and the linear conformation by the yellow diamond. (**c**) NMR structure of chemically synthesized Tv1. Disulfide bonds are depicted in yellow.

SPPS was first developed by Robert Bruce Merrifield in the second half of the twentieth century and has become a standard synthesis method for both peptides and proteins [112]. Through SPPS venom peptides can be rapidly synthesized, allowing the incorporation of unnatural amino acids and peptide backbone modification. The SPPS initiates on the carboxylic end of the last amino acid in a peptide sequence, which is bound to an insoluble solid support or resin. In this technique, a three-step deprotection, activation, and coupling process is repeated until the peptide of interest is completed, at which point it is removed by cleavage from the solid support resin (Figure 5A). The insoluble nature of the resin allows excess reagent to be used to drive the amino acid coupling reaction to completion, and then all excess is washed away at each step in preparation for the next reaction. In the first SPPS iteration, the amino-terminus of each amino acid is protected from unwanted reaction by an acid labile *tert*-Butyloxycarbonyl (BOC) group. In the past few decades several solid support resins have been developed, as well as the now widely used base-labile fluorenylmethyloxycarbonyl (FMOC) amino-terminus protecting group for amino acids [113,114]. The FMOC protecting group is removed or deprotected with a strong base and the next amino acid is activated and then added to the growing peptide chain (Figure 5A). Activation facilitates the coupling reaction and a peptide bond is formed between the amino acid residues (Figure 5A).

Venom peptides, which are typically rich in cysteines, pose several challenges for SPPS. However, most of these challenges can be overcome by using a copolymer solid support that contains both polystyrene and polyethylene glycol. The polystyrene and polyethylene glycol copolymer has greater stability in acidic environments, higher swelling, and prevention of racemization, which is a concern for any peptide sequence with multiple cysteines or histidines [115]. Another strategy to successfully synthesize disulfide rich peptides is to increase purity and yield by incorporating pseudoproline dipeptides to reduce β-sheet formation during synthesis [116]. Typically, cysteine residues are orthogonally protected using an acetamidomethyl group on select cysteines, and trityl groups on the remaining cysteines to allow for site-specific deprotection [117]. More recently substituting select cysteines for selenocysteines [113,118] significantly advanced the synthesis and folding of cysteine rich venom peptides. SPPS has been the method of choice for the synthesis of several conotoxins and also for incorporation of unnatural amino acids such as d-amino acids [43,64,110,111,112,113]. We have recently applied SPPS to successfully synthesize Tv1, a 23-amino acid teretoxin from *Terebra variegata* [72] (Figure 5B,C).

### 4.2. Recombinant Synthesis

Recombinant expression techniques are a great alternative for the synthesis of peptides that are problematic for SPPS due to length or complexity, such as many teretoxins, which can have a length of up to 70 amino acids. Recombinant expression in *Escherichia coli* is a well-established and popular method for the production of recombinant proteins in which the gene of interest is cloned in an expression vector, transformed into the host, and induced, providing a protein product ready for purification [119]. There have been several examples published in the literature describing methodologies for recombinant expression of disulfide-rich peptides [108,120,121,122,123,124,125,126,127,128,129,130]. These studies highlight important aspects that must be considered for recombinant expression of peptides, including the choice of a fusion tag, purification method, host species and strain, and cleavage technique. For example, conotoxin MVIIA from *Conus magus*, was successfully expressed through a recombinant methodology using a thioredoxin *N*-terminal fusion tag, a His-tag for purification, and a BL21 (DE3) *E. coli* host without any cleavage of the fusion tag [120]. Another conotoxin, PrIIIE from *Conus parius*, was recombinantly expressed in a similar way, but a small ubiquitin-like modifier (SUMO) was used as an *N*-terminal fusion tag, Rosetta-gami B (DE3) was used as the *E. coli* host, and the fusion tag was cleaved using SUMO protease [127].

We recently described a method for the recombinant expression and characterization of terebrid teretoxin peptide Tgu6.1, from *Terebra guttata* [111]. The teretoxin Tgu6.1 is a novel 44-amino acid teretoxin peptide with a cysteine scaffold similar to the VI/VII framework (C-C-CC-C-C) of the I, M, and O-superfamilies found in cone snails. The recombinant Tgu6.1 was synthesized using a ligation independent cloning strategy with an ompT protease-deficient strain of *E. coli*. Specific care in plasmid design was taken to combat challenges commonly associated with recombinant expression, such as the formation of insoluble protein aggregates in *E. coli*, proteolytic degradation, and unfavorable conditions in *E. coli* cytoplasm that can prevent the formation of disulfide bonds. In the case of Tgu6.1, thioredoxin was introduced in the plasmid for disulfide folding and solubility issues, His6-tag and Ni-NTA (nickel-nitrilotriacetic acid) affinity chromatography were used as a purification method, and enterokinase was applied to site-specifically cleavage Tgu6.1 from the fusion protein. The recombinantly expressed Tgu6.1 peptide exhibited bioactivity, displaying a paralytic effect when tested in a bioassay using the native prey or terebrids, *Nereis virens* (Annelida) [111].

As the demand for therapeutic peptide drugs increases it is crucial to have reliable methods for obtaining significant amounts of disulfide-rich venom peptides. The recombinant expression technique applied to Tgu6.1 described above is an effective alternative to SPPS of teretoxins and other disulfide-rich venom peptides.

## 5. Characterization of Teretoxin Structure

Determining disulfide connectivity in venom peptides is a fundamental step in establishing structure–function relationships. The disulfide crosslinks in venom peptides provide the structural scaffolds that are essential for their recognition at specific receptor sites [131,132]. An important aspect to determine disulfide connectivity is the ability to sequester fragments containing single disulfide bonds through MS fragmentation [133]. As most venom peptides are highly disulfide-rich, the number of disulfide bond isomers rapidly increases with the number (n) of disulfide bonded Cys residues: the general formula being n!/[(n/2)!2^n/2^]. Traditionally, the determination of disulfide pairing in proteins/peptides was extremely labor-intensive, applying separation of proteolytic fragments by electrophoresis in one dimension, followed by performic acid oxidation and paper chromatographic separation in the other [134]. A theoretically ideal method to determine disulfide frameworks in venom peptides is X-ray crystallography as the dense sulfur atoms in the cysteine side chains scatter electrons well and are therefore readily visible in electron density maps. Unfortunately, the inherent flexibility and small size of most venom peptides make them difficult to crystallize [133,135]. The most commonly used methods for characterization of disulfide bonds involve selective reduction and alkylation of the peptide at low pH followed by Edman sequencing of a panel of partially reduced intermediates or cleavage of the peptide with proteolytic enzymes followed by isolation and MS/MS analysis of the resulting fragments [136,137].

### 5.1. Characterization of Teretoxin Disulfide Motif

We have recently determined the disulfide connectivity of teretoxin Tv1 from *Terebra variegata* by MS/MS mapping using a partial reduction and dual alkylation protocol applying TCEP-HCl (Tris(2carboxyethyl)phosphine hydrochloride) as reducing agent and NEM (*N*-ethylmaleimide) and IAM (iodoacetamide) as alkylating agents. Dual NEM/IAM alkylation resulted in Tv1 peptide species that were labeled with two, four, or six NEM and IAM groups. The location of NEM and IAM modifications in each of the six partially reduced species was determined by matching the MS/MS *b*- and *y*-series ions to theoretical patterns [72].

The solution structure of Tv1 was independently derived using standard homonuclear proton NMR techniques on unlabeled folded synthetic peptide to confirm the disulfide bond connectivity derived from MS/MS (Figure 5C). Proton assignments were obtained from 2D NOESY and TOCSY spectra, and carbon chemical shifts were assigned with the help of a natural-abundance ^13^C-HSQC spectrum. Disulfide connectivities were then determined based on the proximity of cysteine residues in the 10 lowest-energy structures and were in agreement with the disulfide bond pattern derived by MS/MS analysis [72]. Teretoxin Tv1 has a unique fold compared to other venom peptides. The Cys7 to Cys16 β-hairpin is clamped together, and the N- and C-terminal loops are clamped through the Cys4-Cys20 and Cys5-Cys21 double-disulfide bond arrangement in an antiparallel manner that flattens the peptide into an ellipsoid shape (Figure 5C).

### 5.2. *In Silico* Peptide Structure Determination

**Figure 6:**
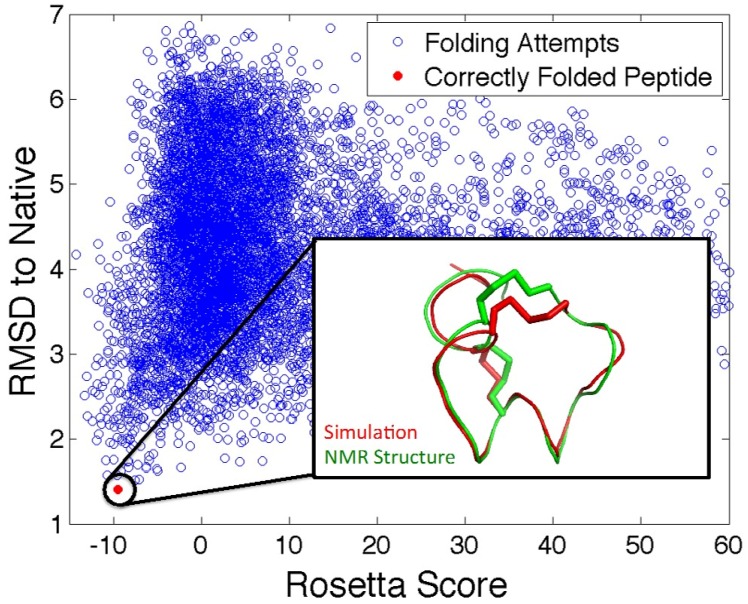
Predicting 3D structure of venom peptides. Scatter plot representation of Rosetta scores for each of the 10,000 attempts to fold α-GID conotoxin from its amino acid sequence. Blue circles represent each folding attempt and the red circle represents a folding simulation that resulted in the correct structure. Inset: comparison of α-GID NMR structure (green) and Rosetta structure prediction (red). Rosetta *ab initio* folding protocol was used to predict structure and scores were calculated as the Root-Mean-Square Deviation (RMSD) to the NMR structure of α-GID.

With the large numbers of putative venom peptides identified recently through NGS approaches, it is prohibitively time-consuming and expensive to structurally characterize each of these peptides using NMR, especially considering that some of the identified peptides might be false positives that represent artifacts of the NGS assembly methods. Bioinformatics algorithms that predict the three-dimensional structures of peptides can be used to narrow down which of many candidate peptide toxins are worthy of experimental characterization [138] (Figure 6). Venom peptide sequences that fold into three-dimensional structures with high confidence are more likely choices for structural and experimental characterization than those that do not form stable folds or display many conformations with no clear global minimum. Using this *in silico* approach, the number of peptides that are synthesized and characterized could be significantly reduced to a manageable amount.

The Rosetta algorithm for protein folding has enjoyed considerable success in accurately predicting the three-dimensional structure of proteins *ab initio* from their sequence, including the prediction of a completely new protein fold [138,139]. Rosetta is well suited for the folding of venom peptides from their primary sequence as the disulfide connectivity of these peptides significantly reduces the number of conformations that need to be searched. Even with this constraint, there is evidence that a very large amount of sampling will be necessary for accurate structure prediction, as venom peptide conformations are unusual in that they differ from the typical, globular conformations of most proteins [140]. Compared to simpler sequence based approaches that neglect to consider information about the three-dimensional structure of venom peptides, Rosetta can be used as a more robust filter for screening the more than one million estimated conoidean venom peptides identified using venomics.

## 6. Teretoxins Bioactivity Assays and Functionalization

Our integrative venomics strategy follows a funneling approach from organismal to molecular biology, starting with the description of terebrid venomous lineages and the characterization of teretoxin peptides, and ending with the identification of specific molecular targets and functions (Figure 1). In the sections below we describe our particular methodology to investigate teretoxin bioactivity and molecular targets.

### 6.1. Biological Assays

To determine the biological activity of selected synthesized teretoxins, peptides are initially tested on a bioassay using their native prey, polychaete worms (Annelida) (Figure 1). Animal assays have proven very useful to gain initial phenotypic insight in to the function of conoidean peptides [141]. Teretoxin polychaete assays are conducted by injecting the folded terebrid peptide in the ventral nerve cord of a polychaete. Two additional polychaetes are usually injected with saline solution as a negative control, and a well-characterized peptide toxin (e.g., agatoxin) as a positive control [39,72,111]. Two recently characterized teretoxins, Tv1 and Tgu6.1, analyzed using this bioassay caused partial paralysis in *Nereis virens* polychaetes [72,111]. As Terebridae native prey, polychaete worms are the first line of attack to determine bioactivity in terebrid venom peptides. More complex animal assays, such as rat or mouse models, are the next step and routinely used to assay venom activity [142,143,144,145,146,147]. While functionality and activity in native prey are not directly tied to drug discovery, conducting native prey assays ensures that the newly identified peptide is synthesized and folded correctly. It is important to verify that the peptide scaffold being applied for drug development is an accurate scaffold. Additionally, screening for venom peptide molecular targets and bioassays for potential biomedical applications is very labor-intensive, so focusing on peptides that show bioactivity in native prey narrows the pool of candidates to those that have greater potential. Finally, the phenotypic response in the native prey can help identify the molecular mechanism of the venom peptide, e.g., if it shows paralytic effect or hyperactivity this may suggest a possible molecular ion channel target based on previous peptides screened. More recently, due to the increasing interest in venom peptides as candidates for drug discovery, microfluidic techniques using cell cultures are also being applied to assay crude venom extracts and purified peptide toxins [148,149]. One of the main advantages of microfluidics is that it allows for fast high-throughput screening of venom peptides to rapidly identify bioactive compounds and their potential molecular targets.

### 6.2. Characterizing Molecular Function

Venom peptides typically interact with ion channels and modulate their activities, enabling the investigation of specific ion channels and their function [150,151]. For that purpose, after bioactivity is confirmed through a phenotypic screen, the next step is to determine the molecular target of the peptide toxin. Characterizing the molecular activity of venom peptides is important from a basic scientific perspective, but also critical from a therapeutic point of view, as knowing the mechanism of action of a molecule is a prerequisite for moving it through clinical trials. Additionally, identifying the molecular site of action of venom peptides enables an ensemble of structure-based molecular design methods to optimize the peptides use as effective drugs. However, identifying the receptors on which a venom peptide is active can be as difficult as finding a needle in a haystack.

Virtual screening is a well-established computational method for identifying ligands that interact with target proteins. It has been applied successfully even to challenging problems such as finding small molecules that are active at a target protein whose structure is not known, such as a G-Protein Coupled Receptors (GPCRs) [152]. In theory, virtual screening methods could also be used to identify the molecular targets of a newly discovered venom peptides. However, in practice this could be challenging due to the laborious nature of constructing individual models of venom peptides with a variety of different potential molecular targets—such as nicotinic receptors, voltage-gated sodium and calcium channels, and Transient Receptor Potential (TRP) channels—and those for which there is no solved NMR or crystal structure of the peptide or molecular receptor.

Molecular modeling environments that integrate bioinformatics, homology modeling, and docking algorithms can drastically reduce the time needed to create *in silico* venom peptide models. In this regard, virtual screening can be used in conjunction with high-throughput bioassay screening methods to prioritize which molecular targets to screen against. For example, the Bioluminate software package (Schrodinger; New York, NY, USA) largely automates all the steps in the homology model process, including special features that allow more sensitive searches for distant structural homologues of ion channels to use as templates. The entire homology modeling process takes only a few minutes. The venom peptide can then be docked against the model using the integrated PIPER protein–protein docking algorithm. The entire process, including simulation time, takes roughly one hour for a given ion channel or receptor target. While the results of such an *in silico* screen may not always be entirely accurate, they can be improved by including mutagenesis constraints from the literature if available. Such an effort can thus provide a prioritized list of molecular targets for screening (*i.e.*, start with sodium channels prior to calcium channels), potentially reducing the time and material necessary to identify the molecular channels targeted by venom peptides.

Our approach to teretoxin molecular target discovery is to apply computational algorithms to model the docking of the peptide of interest to a wide range of potential receptors. The docking poses can be refined with long timescale Molecular Dynamics (MD) simulations of the peptide toxin/receptor pose. If the peptide remains in a well-defined pose over the timescale of hundreds of nanoseconds or several microseconds, it suggests that the teretoxin effectively binds the target receptor protein. These receptors are then selected as the more likely candidates and have the highest priority for further experimental verification. Alternatively, receptors where the peptide never establishes a well-defined pose are considered less likely to be the true target of the peptide and are discarded for experimental testing.

## 7. Optimization of Venom Peptides for Drug Development

The estimate of available venom peptides from the reservoir of conoidean snails alone is upwards of one million compounds [19]. Giving this enormous grab bag, it is essential to identify methods for optimizing the selection of venom peptides for drug development. Prialt^®^, Byetta^®^, and Captopril^®^ are all breakthrough drugs derived from animal venom peptides via different routes, decades after their initial discoveries [6,7,8]. However, with the promise of venomics, peptides that lead to therapeutics can be more effectively identified in a strategic manner. It should be noted that venom peptides, while more stable due to their disulfide-rich content, are still susceptible to hurdles that prevent their widespread application as therapeutics, namely poor pharmacokinetics and invasive delivery methods [153,154]. The sections below outline the strategies that we apply to optimize the potential biomedical applications of teretoxins.

### 7.1. Computational Design for Increased Affinity and Selectivity of Peptide Toxins

Native venom peptides have remarkable affinity and specificity for drug targets such as ion channels and GPCRs. Many venom peptides can readily serve as scaffolds for peptidomimetics or pharmacological research tools; however, with a few exceptions, most venom peptides often require derivative versions to be useful as therapeutic leads. A common modification applied to venom peptides is devising derivatives to increase affinity for a specific molecular receptor [155,156]. Another modification often required for peptide toxins is cyclization to increase the potential for oral activity and longevity for *in vivo* circulation [157,158,159,160].

Traditional methods for identifying specific functional mutations in venom peptides include trial and error alanine walks through each residue of the peptide. Following this approach, 20 potential functional mutations in conotoxin α-GID and 70 mutants of spider peptide GpTx-1 were identified [161,162]. However, there is no guarantee that any of these alanine mutants will have the desired pharmacological profile [163]. Moreover, alanine scans followed by synthesis and characterization of each mutant is costly and time-consuming. A modern alternative to alanine scans involves bioinformatics algorithms in which different mutations to the peptide toxin can be applied *in silico* and their effects on affinity and selectivity of binding to specific receptors can be predicted computationally [164,165]. The *in silico* method is both inexpensive and rapid compared to alanine scans, ensuring that the number of venom peptides examined can be significantly increased.

**Figure 7:**
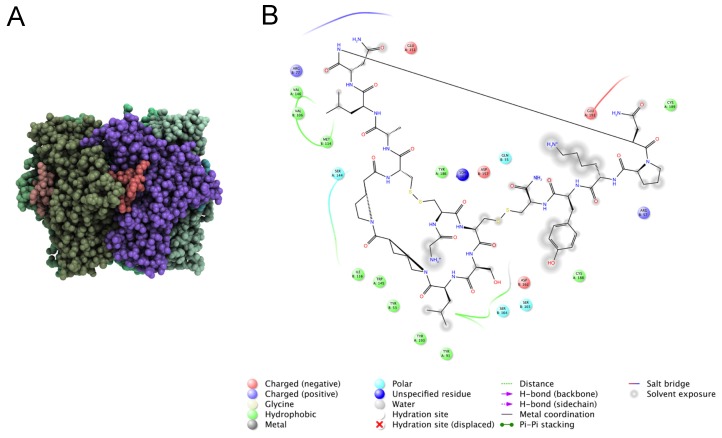
Structure-guided design of venom peptides. (**A**) Structure of acetylcholine binding protein (AchBP) in complex with conotoxin α-PnIA. AchBP subunits (green and purple) have a pentameric arrangement around a central pore. Conotoxin α-PnIA (red) binds at the interface of consecutive subunits. (**B**) Atomic interactions between α-PnIA at the interface of AchBP subunits. Hydrophobic interactions (green) are highly prevalent, but positive and negative interactions are also present. The AchBP binding pocket is extensively exposed to solvent (gray clouds) complicating the computational modeling.

Rosetta is one of the most widely used and successful algorithms for *in silico* molecular design [139]. In addition to being used for modeling the 3D structure of proteins, as discussed previously, Rosetta can also be applied to model and design peptide/receptor complexes, including modules for structural refinement, protein–peptide docking, and protein design [166,167,168]. Additionally, Rosetta has recently been extended to incorporate non-canonical amino acids, therefore it can also model and design post-translational modifications such as, hydroxylation, sulfation, and others commonly found in venom peptides [105,169]. We are currently using Rosetta to increase conotoxin and teretoxin selectivity for specific molecular targets (Figure 7). As part of this effort, we are developing an application inside the Rosetta framework to more accurately predict peptide toxin affinity and specificity by incorporating the flexibility of the peptide/receptor complex into the scoring calculation. When complete, this tool will be made publicly available via Rosetta’s webserver ROSIE [170].

### 7.2. Identification of Key Residues in Venom Peptides

Rosetta on its own will identify potential residues that can be altered to enhance venom peptide specificity. However, if we provide Rosetta with the information accumulated through millions of year of evolution inherent to venom peptide genetic sequences, we can significantly boost its efficiency. Computational algorithms that estimate sequence evolution such as PAML (Phylogenetic Analysis by Maximum Likelihood) and HyPhy (Hypothesis testing using Phylogenies), can compute the rate of non-synonymous to synonymous mutations in a given group of sequences, identifying specific sites of the venom peptides under positive selection [171,172,173]. As these sites are not evolutionarily conserved, with diverse amino acids present in different bioactive peptides, they represent excellent targets to mutate *in silico* with Rosetta. By combining Rosetta modeling with evolutionary algorithms, we can optimize the process of identifying random mutation possibilities, focusing only on those that have passed the test of millions of years of evolutionary change while maintaining venom peptide bioactivity.

In venom research, evolutionary algorithms have been primarily used to answer questions about venom peptide evolution [174,175,176,177,178]. Venoms are generally under strong positive selection to counteract the evolving defenses of their prey in a never-ending predator-prey arms race [179,180,181]. Although traditionally used to investigate evolutionary patterns, evolutionary algorithms can also be effectively applied to predict which amino acids can be altered to increase the affinity and selectivity of a venom peptide to its target [182,183,184]. For example, PAML was successfully used to identify four positively selected sites in scorpion α-neurotoxins LqhαIT, Lqh2, Amm8rgp-3, Ac1, Ac4, Lqh3.1, and Bjα2 that target voltage-gated sodium channels [182]. Two of the four sites identified by PAML as being positively selected had been previously linked to bioactivity in peptides LqhαIT and Lqh2. Additionally, after mutagenesis analysis of these positions, the peptides displayed enhanced potency and selectivity for sodium channels [182,183,184]. Conversely, another study used similar methods to investigate evolutionary patterns in scorpion α-neurotoxin receptors, namely the sodium channels of the scorpion’s prey, and discovered that scorpion venom peptides bind to evolutionarily variable regions of the sodium channels [185]. Specifically, positively selected sites of scorpion α-neurotoxins bind to sodium channels sites under relaxed purifying selection [185]. These findings highlight how venom peptides interact with their molecular targets and indicate specific sites of the peptide and receptor that could potentially be altered to increase selectivity. Therefore, information derived from evolutionary algorithms such as PAML and HyPhy can be coupled with Rosetta software to effectively enhance its predictive properties and increase venom peptide and receptor specificity.

## 8. Venom Peptide Drug Delivery

**Figure 8:**
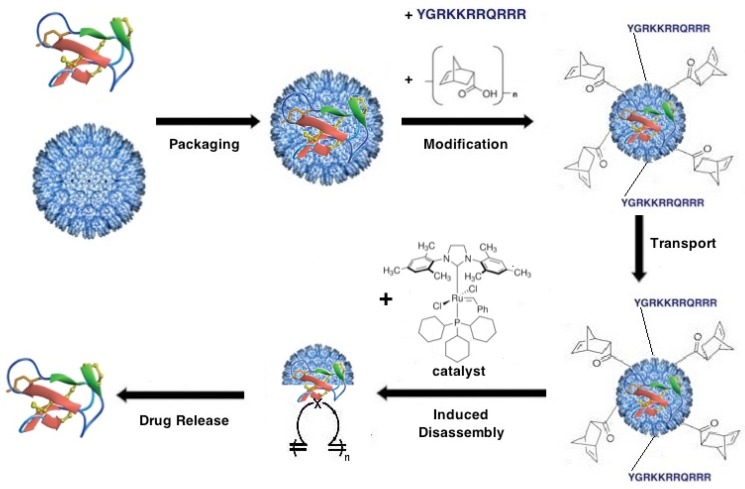
Trojan Horse teretoxin delivery strategy. Schematic overview of peptide drug delivery via virus-like particle (VLP) nanocontainers. The peptide cargo is first encapsulated in the VLP using recombinant biology. The VLP exterior is modified with the cell-penetrating peptide HIV-Tat and norbornene to enable transport to target site and disassembly respectively. The modified VLP nanocontainer is transported to the target site, disassembly is triggered by Grubbs II catalyst and the peptide cargo is released. The modular strategy outlined allows for substitution of alternate conjugates, cargo proteins, and disassembly mechanisms.

The potency and specificity of bioactive peptides have propelled these agents to the forefront of pharmacological research, but delivery of peptides to their molecular target is a major obstacle to their widespread application. We have recently devised a Trojan Horse strategy consisting of packaging a bioactive peptide within a modified protein cage to protect it during transport, and releasing it at the target site, which has proven to be a very promising delivery method [186] (Figure 8).

As mentioned earlier, a major obstacle to the medical application of molluscan venom peptides, and indeed peptides in general, is their poor pharmacokinetic profile. In addition, peptides generally exhibit poor membrane solubility and can be rapidly cleared through the liver and kidneys [154]. Finally, the blood–brain barrier (BBB) prevents neuroactive peptides in the bloodstream from reaching targets in the central nervous system (CNS), resulting in these compounds being administered through intrathecal injection [187]. General methods for improving the pharmacokinetic profile of bioactive peptides are necessary if these compounds are to realize their full therapeutic potential.

There are numerous strategies for improving the pharmacokinetic profile of therapeutic peptides. One approach is to stabilize the structure of the peptide itself through such methods as peptide stapling, macrocyclization, or grafting of peptide segments onto a small protein scaffold [157,158,159,160,188]. However, as these methods involve changes in secondary and tertiary structure, they can disturb the function and bioactivity of the peptide. In addition, these methods are not completely general and must be specifically adapted to each individual peptide.

As an alternative to modifying the peptide, our Trojan Horse strategy involves packaging the peptide of interest within a macromolecular nanoparticle that can deliver it to its molecular target and protect it from degradation during transport. Several types of macromolecules have been investigated as potential drug-delivery nanocontainers including liposomes [189], natural and synthetic polymers [190], inorganic particles [191,192], DNA origami structures [193], and protein cages such as ferritins and virus-like particles (VLPs) [194,195]. Nanoparticle delivery systems are essentially modular, because their packaging, delivery, and targeting properties are determined by the nanoparticle carrier rather than by the therapeutic compound. As a result, a single delivery system could be used for the delivery of a diverse array of bioactive venom peptides.

### 8.1. P22 Nanocontainers for Venom Peptide Drug Delivery

Our recently developed peptide drug delivery method repurposes the procapsid from the *Salmonella typhimurium* bacteriophage P22 as a nanocontainer for the delivery of ziconotide (Prialt^®^, MVIIA) across the blood-brain barrier (BBB) [186]. Similar to other viral capsids, the P22 procapsid is well-defined, monodisperse, easy to manufacture, and amenable to both chemical and genetic manipulation [196]. By modifying the scaffold protein that templates the self-assembly of the P22 procapsid, an arbitrary gene product can be incorporated within the procapsid shell [197,198]. Among the proteins that have been successfully packaged within the procapsid are the fluorescent proteins EGFP, mCherry, and ziconotide [186,197,199].

VLPs have a number of significant advantages compared with other macromolecules: First, they are generally uniform in size and composition and possess defined architectures—traits that can allow for precise control of pharmacological properties. Second, as proteins, they are biodegradable by endogenous cellular pathways, reducing the ability to accumulate in an organ. Also, as gene products, VLPs can be produced relatively easily and in high yields using standard molecular biology protocols. Finally, a plethora of tried-and-tested protein modification techniques are available for manipulating the interior and exterior of proteinaceous VLPs such as molecular cloning, standard and unnatural amino acid mutagenesis, protein bioconjugation, and directed evolution [200].

In general, the adaptation of protein cages for drug delivery involves three distinct steps: (1) encapsulation of the pharmacological agent within the viral capsid; (2) targeting of the capsid to the desired site *in vivo*; and (3) induced disassembly of the capsid and release of the cargo under physiological conditions (*i.e.*, neutral pH, moderate temperature, and aqueous environment) (Figure 8). Applying our Trojan Horse strategy, we have successfully transported P22 VLPs loaded with the conotoxin-derived analgesic ziconotide (Prialt^®^), across *in vitro* and *in vivo* BBB models. Briefly, the cell-penetrating HIV-Tat peptide (YGRKKRRQRRR) was synthesized, fluorescently labeled, and activated with maleimidopropionic acid (MPA), then conjugated to a P22 nanocontainer preloaded with ziconotide and engineered to feature a surface exposed cysteine residue. P22-Tat nanocontainers translocated the BBB, demonstrating the feasibility of this Trojan Horse strategy [186]. At a size of ~54 nm in diameter, P22 capsid virus-like particles are significantly larger than the proteins and quantum dots previously translocated and reported in the literature. This was the first demonstration of delivery of ziconotide across a BBB model using a nanoparticle delivery system, providing an alternative route to intrathecal injection, which has thus far been the only delivery method. The results of this proof-of-concept experiment are promising towards the development of a tunable VLP nanocontainer for the delivery of peptide therapeutics across the BBB [186,199].

### 8.2. Release of Venom Peptide at Molecular Target Site

The next step in the adaptation of VLPs for drug delivery, namely controllable disassembly, remains a challenge, mainly due to the fact that the disassembly mechanism must proceed under physiological aqueous environment, with moderate temperature and neutral pH. Overcoming this obstacle requires an integrated approach at the intersection of chemistry and biology, with a particular emphasis on materials science, structural virology, and protein engineering. Recent advances in bioorthogonal chemistry have led to the identification of numerous reactions that proceed under physiological conditions. We are currently investigating the Ring Opening Metathesis Polymerization (ROMP) triggered by a ruthenium catalyst (Grubbs II catalyst) for the controlled disassembly of the P22 VLP (Figure 8).

ROMP is a polymerization reaction initiated by a transition-metal catalyst and driven by the release of ring strain in a cyclic olefin such as cyclobutene, cyclopentene, *cis*-cyclooctene, or norbornene [201]. The ROMP disassembly strategy aims to disrupt the capsid architecture through steric strain brought about by the unfolding polymerization reaction [202,203,204]. While most ROMP catalysts, including all of the commercially available catalysts, use ruthenium, ROMP with molybdenum and tungsten catalysts have also been reported [205]. As we have previously demonstrated, norbornene is readily attached to the surface of the P22 procapsid through traditional bioconjugation techniques [186,199]. We have recently found that conjugation of P22-GFP procapsids with NHS-activated 5-norbornene-2-carboxylic acid yields P22-GFP-Norb procapsids with an average of 4.12 norbornenes per coat protein monomer, or more than 1700 norbornenes per procapsid [199]. While the coat protein monomer contains 19 lysine residues, not all of these are surface exposed. TEM analysis revealed that conjugation of norbornene to the capsid surface did not significantly affect size or morphology. However, treatment of P22-GFP-Norb with Grubbs II catalyst, which initiates ROMP, produced clusters of P22-GFP-Norb with distorted morphologies that appeared to be joined by robust bridge structures, suggesting that ROMP occurred at both intra- and inter-nanocontainer interfaces [199]. Further investigation remains to be done to characterize the triggered disassembly of P22 VLPs, but these early results suggest that our Trojan Horse strategy is an effective method for the delivery of peptide therapeutics, including teretoxins.

## 9. Conclusions

This review describes our *learn-from-nature* integrative venomics strategy for the discovery and characterization of terebrid venom peptides (Figure 1). This multidisciplinary strategy from mollusks to medicine starts with the phylogenetic delimitation of venomous Terebridae lineages and putative teretoxins, continues with the chemical and recombinant synthesis of promising peptide toxins and their structural characterization, followed by assays to determine bioactivity and molecular targets, and concludes with the optimization of venom peptides as drug leads and the development of effective strategies for delivery of venom peptide therapeutics. While a significant amount of research remains to be done, it is clear that venoms are nature’s cocktail for drug discovery and an integrated venomics strategy is a successful route to identifying the most effective ingredients to develop potent and selective peptide therapeutics.
